# Onchocerciasis: The Pre-control Association between Prevalence of Palpable Nodules and Skin Microfilariae

**DOI:** 10.1371/journal.pntd.0002168

**Published:** 2013-04-11

**Authors:** Luc E. Coffeng, Sébastien D. S. Pion, Simon O'Hanlon, Simon Cousens, Adenike O. Abiose, Peter U. Fischer, Jan H. F. Remme, K. Yankum Dadzie, Michele E. Murdoch, Sake J. de Vlas, María-Gloria Basáñez, Wilma A. Stolk, Michel Boussinesq

**Affiliations:** 1 Department of Public Health, Erasmus MC, University Medical Center Rotterdam, Rotterdam, The Netherlands; 2 UMI 233, Institut de Recherche pour le Développement (IRD), University of Montpellier 1, Montpellier, France; 3 Department of Infectious Disease Epidemiology, School of Public Health, Faculty of Medicine (St Mary's Campus), Imperial College London, London, United Kingdom; 4 Department of Epidemiology and Population Health, London School of Hygiene and Tropical Medicine, London, United Kingdom; 5 Sightcare International, Secretariat Main Office, Ibadan, Oyo State, Nigeria; 6 Washington University School of Medicine, Infectious Disease Division, St. Louis, Missouri, United States of America; 7 Bernhard Nocht Institute for Tropical Medicine, Hamburg, Germany; 8 Independent Consultant, Ornex, France; 9 Independent Consultant, Accra, Ghana; 10 Department of Dermatology, Watford General Hospital, Watford, Hertfordshire, United Kingdom; London School of Hygiene and Tropical Medicine, United Kingdom

## Abstract

**Background:**

The prospect of eliminating onchocerciasis from Africa by mass treatment with ivermectin has been rejuvenated following recent successes in foci in Mali, Nigeria and Senegal. Elimination prospects depend strongly on local transmission conditions and therefore on pre-control infection levels. Pre-control infection levels in Africa have been mapped largely by means of nodule palpation of adult males, a relatively crude method for detecting infection. We investigated how informative pre-control nodule prevalence data are for estimating the pre-control prevalence of microfilariae (mf) in the skin and discuss implications for assessing elimination prospects.

**Methods and Findings:**

We analyzed published data on pre-control nodule prevalence in males aged ≥20 years and mf prevalence in the population aged ≥5 years from 148 African villages. A meta-analysis was performed by means of Bayesian hierarchical multivariate logistic regression, accounting for measurement error in mf and nodule prevalence, bioclimatic zones, and other geographical variation. There was a strong positive correlation between nodule prevalence in adult males and mf prevalence in the general population. In the forest-savanna mosaic area, the pattern in nodule and mf prevalence differed significantly from that in the savanna or forest areas.

**Significance:**

We provide a tool to convert pre-control nodule prevalence in adult males to mf prevalence in the general population, allowing historical data to be interpreted in terms of elimination prospects and disease burden of onchocerciasis. Furthermore, we identified significant geographical variation in mf prevalence and nodule prevalence patterns warranting further investigation of geographical differences in transmission patterns of onchocerciasis.

## Author Summary

Until recently, elimination of onchocerciasis (river blindness) from Africa by mass treatment with ivermectin alone was deemed impossible. However, recent reports of elimination of onchocerciasis from various African foci have stimulated renewed interest. An important determinant of achieving elimination is the pre-control microfilarial (mf) prevalence, i.e. the percentage of people with larval stages of the *Onchocerca volvulus* worm in the skin, which can be detected in a skin snip (a small skin biopsy). Because this method is considered invasive, pre-control infection levels in Africa have been mapped mostly by means of palpation of subcutaneous nodules (protuberances under the skin where the adult worms live) in adult males, a relatively crude but non-invasive method of detecting infection. We developed a tool to derive estimates of pre-control mf prevalence from available pre-control nodule prevalence estimates. This tool can help evaluate ongoing control programs, help assess local elimination prospects, and help estimate levels of disease due to onchocerciasis by linking pre-control nodule palpation data to the large body of literature on the association between mf prevalence and disease.

## Introduction

In 1995, the World Health Organization launched the African Programme for Onchocerciasis Control (APOC). At that time, APOC aimed to control morbidity due to onchocerciasis (river blindness) in Africa, with a focus on those countries not covered by the previous Onchocerciasis Control Programme in West Africa (OCP). Since 1995, APOC has successfully coordinated mass treatment with ivermectin in sixteen onchocerciasis-endemic African countries [1]. Until recently, elimination of onchocerciasis from African foci was deemed to be not achievable by means of mass ivermectin treatment alone, considering the large size of the transmission zones, the mobility of the insect vectors and human populations, and poor compliance with mass treatment in some areas [2]. However, following the first reports of elimination of onchocerciasis from foci in Mali, Senegal, and Nigeria by mass treatment alone [3], [4], [5], there is renewed interest in elimination of onchocerciasis from Africa [6].

Pre-control infection levels are an important predictor of morbidity levels [7], [8], [9] and the duration of onchocerciasis control programs required to achieve elimination of infection [10], [11]. High pre-control levels of infection indicate circumstances that are favorable for intense transmission in terms of vector abundance, proximity to vector breeding sites, high vectorial capacity and competence, etc. In such circumstances, mass treatment with a drug such as ivermectin, which is predominantly microfilaricidal, but has a lesser impact on adult worm survival, needs to be continued for a long time and at high therapeutic and geographical coverage before it can be stopped without considerable risk of recrudescence of infection. Progress towards elimination of onchocerciasis from APOC areas is currently being evaluated by means of ongoing skin snipping surveys that measure levels of infection in terms of presence and density of microfilariae (mf) in the skin of the general population [5]. In contrast, pre-control levels of infection in APOC areas have been quantified by the REMO method (rapid epidemiological mapping of onchocerciasis), which is based on the palpation of subcutaneous nodules containing adult *Onchocerca volvulus* worms in a sample of 30–50 males aged ≥20 years in villages selected using a standardized selection procedure [12], [13]. Results from pre-control and ongoing surveys will have to be compared, even though the REMO method is much cruder for detecting presence and intensity of infection than skin snipping. Therefore, it is important to assess how informative pre-control nodule palpation data are, and when and whether they can be reliably translated to equivalent measures of skin microfilariae. In other words, there is need for a quantitative model describing the association between pre-control nodule prevalence and pre-control presence of skin microfilariae, which takes into account the differences between the two methods as well as other covariates. Such a model would also allow estimates of pre-control nodule prevalence to be related to the large body of literature on the correlation between mf prevalence and prevalence of onchocercal morbidity, allowing better estimation of the disease burden of onchocerciasis.

We present a statistical model describing the association between pre-control nodule prevalence in adult males and pre-control mf prevalence in the general population. Quantitative relationships for this association have been previously described, but were based on smaller number of surveys, did not provide estimates of uncertainty around parameter estimates and model predictions, and did not account for geographical variation or the relatively small sample sizes routinely used for the nodule palpation method, resulting in attenuation bias (due to measurement error in nodule prevalence) [14], [15], [16], [17]. In this study, we analyzed original pre-control data, accounting for these factors, and using Bayesian statistical methods, well known for providing robust uncertainty estimates around model parameters.

## Methods

### Data and Study Sites

**Figure 1:**
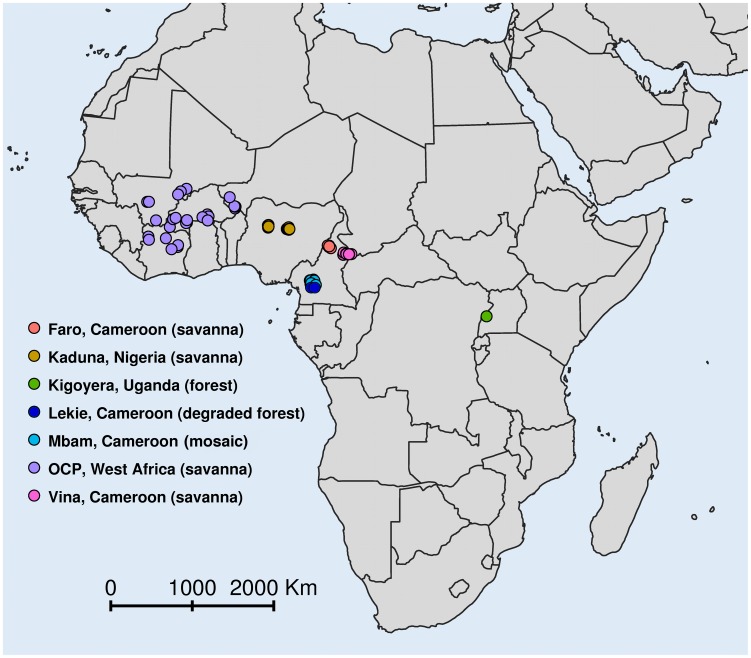
Locations of study sites.

We analyzed original data on pre-control nodule prevalence in adult males (N = 7,525 individuals) and mf prevalence in the population aged five years and above (N = 29,775 individuals) from 148 villages in seven geographical areas including countries in the former OCP area, and foci in Cameroon, Nigeria, and Uganda, which are part of APOC (Table 1, Figure 1). Most of these data have been previously published [9], [14], [18], [19], except for part of the data from Cameroon. The simuliid vectors responsible for transmission in each area have been described previously (Table 1) [9], [19], [20], [21], [22], [23]. In all areas, data on nodule and mf prevalence had been collected simultaneously (except for Nigeria, where nodule palpation took place six to twelve months after skin snipping, though still before the start of control interventions). All data on mf prevalence were based on taking two skin snips (one from each iliac crest) from each individual examined, which were incubated in saline for 24 hours, and village-level prevalence values were age- and sex-standardized according to the reference OCP population (direct standardization, supplementary Table S1). Then, we calculated the standardized number of mf positive persons in a village by multiplying the standardized prevalence with the sample size, and rounding to the nearest integer. Nodule prevalence was based on palpation-based detection of nodules that could be attributed to onchocerciasis with reasonable certainty, similar to the methodology used for mapping of infection in APOC areas; i.e. nodules of uncertain etiology (e.g. possible enlarged lymph nodes) were excluded [12]. All data were used with permission of the authors who originally collected such data, and were analyzed anonymously.

**Table 1:** Characteristics of data used for modeling the association between prevalence of nodules and microfilariae.

Area	Number of villages	Number of males examined for nodules	Number of individuals from general population examined for microfilariae in the skin	Bioclime	Vector responsible for transmission (*Simulium* spp)	Reference
Kigoyera Parish, Uganda	8	667	2,085	Forest	*S. neavei s.s.* [19]	[19]
Onchocerciasis Control Programme in West Africa	26	1,386	5,273	Savanna	*S. damnosum s.s.* and *S. sirbanum* [9]	[9], [14], [18]
Kaduna, Nigeria	33	1,822	7,274	Savanna	*S. damnosum s.s.* and *S. sirbanum* [21]	[14], [18]
Lekié, Cameroon	19	806	3,430	Degraded forest	*S. squamosum B* [23]	unpublished
Mbam, Cameroon	34	1,354	6,190	Forest-savanna mosaic	*S. squamosum B* [23]	unpublished
Vina, Cameroon	19	1,122	4,266	Savanna	*S. damnosum s.s.* and *S. sirbanum* [20]	[14], [18]
Faro, Cameroon	9	368	1,257	Savanna	*S. damnosum s.s.* and *S. sirbanum* [22]	unpublished

*s.s.*: sensu stricto.

### Statistical Methods and Model Fitting

The association between village-level mf prevalence and nodule prevalence was quantified in a meta-analysis by means of hierarchical multivariate logistic regression, i.e. logistic regression where the predicted outcome is a set of correlated binary random variables rather than a single binary random variable. A hierarchical approach was taken to account for unmeasured sources of variation between geographical areas. A multivariate approach was taken to account for measurement error in each measure of infection. This approach prevents regression of model coefficients towards zero (attenuation bias) as we do not have to assume that there is no measurement error in the explanatory variable (e.g. either nodule or mf prevalence), an assumption inherent to univariate regression [24].

We extended the ordinary hierarchical logistic regression model to a multivariate model simultaneously predicting *m* binary outcomes:
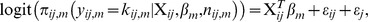
where 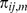 is the probability of finding 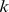 cases of the *m*-th binary outcome (*m* = 1: presence of microfilariae in the skin; *m* = 2: presence of nodules in adult males) among 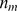 observed individuals from the *i*-th unit (village) and the *j*-th cluster (geographical area). The error terms 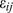 and 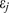 (each consisting of *m* components) represent the variation (random effects) in infection levels within and between the *j* geographical areas, respectively. For each village there is a set of observed covariates 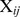, and for each of the *m* predicted binary outcomes there is a set of parameters 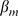 (fixed effects), where the intercepts 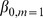 and 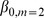 represent the mean log odds of presence of mf in the general population (all those aged ≥5 years) and nodules in adult males. To explain possible large differences between geographical areas related to bioclime, parasite strains and clinical manifestations in onchocerciasis [25], we included a set of coefficients for bioclimatic zone in the model. Here, the parameters 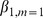 and 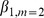 represent the log odds ratio of observing microfilariae in the skin and subcutaneous nodules in forest areas (including degraded forest and forest-savanna mosaic areas), relative to savanna areas. Correlation between nodule and mf prevalence was modeled by assuming a multivariate normal distribution for the *m* components of the error term at each level of analysis. See supplementary Text S1, section “Model description” for a more detailed description of the model.

To account for measurement error due to misclassification of nodules (e.g. classifying lymph nodes as onchocercal nodules due to imperfect specificity; or failing to detect at least one subcutaneous onchocercal nodule when one or more are present, due to imperfect sensitivity), we added parameters to the model for specificity and sensitivity of nodule palpation, allowing these to be estimated from the data. Prior information for parameter values was based on the literature. A wide range of values is reported for specificity (60%–99%), based on various definitions [15], [19], [26], [27]. We assumed that when performed by physicians experienced in recognizing onchocercal nodules, specificity of nodule palpation is between 98% and 100%, based on the report of finding only four non-onchocercal nodules among 312 extirpated nodules [19]. Further, we assumed that sensitivity increases with level of infection, reflecting the notion that detection of at least one nodule is more likely in a person with many onchocercal nodules than in a person with few or only one [27]. In literature, no values for sensitivity of nodule palpation as a method for detecting onchocercal nodules are reported. In the current study, sensitivity was assumed to increase linearly from some unknown minimum sensitivity (value between 60% and 100%) for nodule prevalences close to zero (when persons with nodules have few nodules) to 100% for nodule prevalence of 100%. The choice of a linearly increasing pattern was based on a simulation exercise in which we examined the association between the proportion of the nodule carriers that is detected and the ‘true’ nodule prevalence, given simulated true nodule counts (assuming a negative binomial distribution of counts within a village) and some probability to detect each nodule (minimum sensitivity). A sensitivity analysis showed that the model fit and model predictions did not change when assuming different values for minimum sensitivity of nodule palpation at low infection levels (60%, 80%, or 100%). This is explained by the fact that sensitivity is most important for high prevalence settings (for which we assume sensitivity is high anyway), and far less important in low prevalence settings (where misclassification is largely governed by specificity). Therefore, we simplified the final model by leaving out the parameter for sensitivity, effectively assuming 100% sensitivity of nodule palpation for all infection levels.

Based on the model described above, we estimated the conditional distribution of mf prevalence in a hypothetical village outside the dataset, given an estimate of the ‘true’ nodule prevalence in adult males (i.e. corrected for misclassification of nodules). We assumed that nodule prevalence estimates were based on a sample of 30 adult males, the minimal sample size used in REMO surveys [12], [13]. See Text S1, section “Model application” for a more detailed description of the methods for predicting mf prevalences in hypothetical villages.

The model was fitted to the data in a Bayesian framework. Posterior distributions of parameters and predictions were simulated in JAGS (see Text S1, section “Model specification in JAGS” for code), a program for analysis of Bayesian models using Markov Chain Monte Carlo (MCMC) simulation based on the Gibbs sampling algorithm (version 3.2.0; Martyn Plummer, 2012, http://mcmc-jags.sourceforge.net). Simulations in JAGS were set up and analyzed in R (version 2.14.2) [28], using packages *rjags* (version 3–5, Martyn Plummer, 2011, http://CRAN.R-project.org/package=rjags) and *R2jags* (version 0.03-06, Yu-Sung Su, 2011, http://CRAN.R-project.org/package=R2jags). Improvements in model fit by addition of parameters were assessed via the deviance information criterion (DIC), a generalization of Akaike's information criterion for hierarchical models (lower values indicate better fit, taking into account model deviance and the effective number of parameters in the model) [29]. See Text S1, section “Parameter estimation” for further details about model fitting and checking of model convergence.

The final fit of the model to the data was evaluated by means of mixed posterior predictive checks [30], [31]. In this procedure, the number of individuals positive for mf and nodules in each village was resampled 40,000 times from the estimated joint posterior distribution of model parameters, including resampling of all random effects, and the resulting replicate dataset was compared to the original data.

## Results

The median nodule prevalence in males aged ≥20 years was 58% (range: 2%–100%), and the median mf prevalence in the population aged five years and above was 74% (4%–99%). The median sample size for nodule prevalence in a village was 42 (range: 9–181). The median sample size for mf prevalence in a village was 167 (33–727).

**Figure 2:**
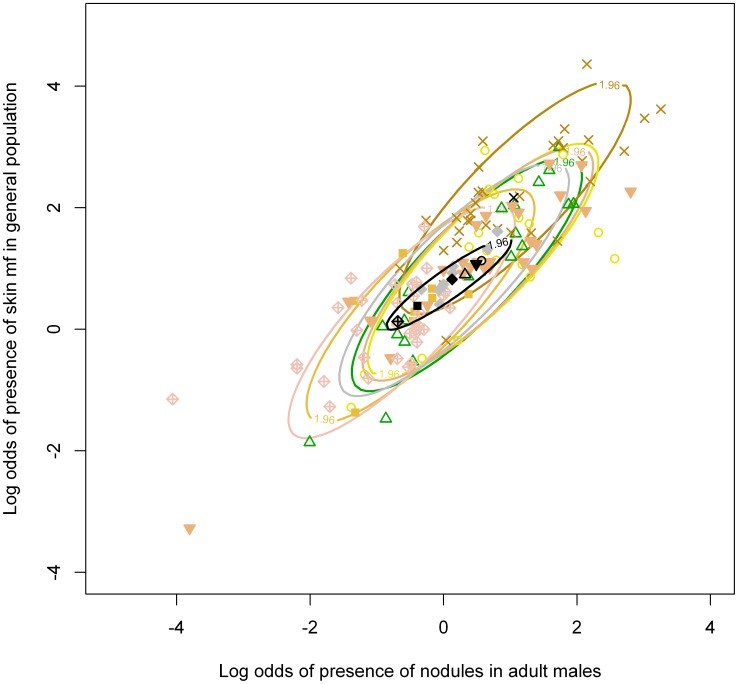
Association between prevalence of nodules in adult males and skin mf in the general population. Colored symbols represent data from seven geographical areas. Colored ellipses indicate the 95% percentiles (Z = 1.96) of the predicted joint distributions of infection prevalences within each geographical area, based on the estimated variances and correlation of observations within geographical areas. Black symbols represent the mean infection prevalences in each of the geographical areas. The black ellipse represents the 95% percentile of the joint distribution of mean infection prevalences in geographical areas, illustrating the deviating pattern in nodule and mf prevalence in Mbam, Cameroon (black and brown crosshairs and brown ellipse). Predictions were based on a Bayesian hierarchical multivariate logistic regression model with a fixed effect for Mbam, Cameroon, and random effects for other geographical areas.

**Figure 3:**
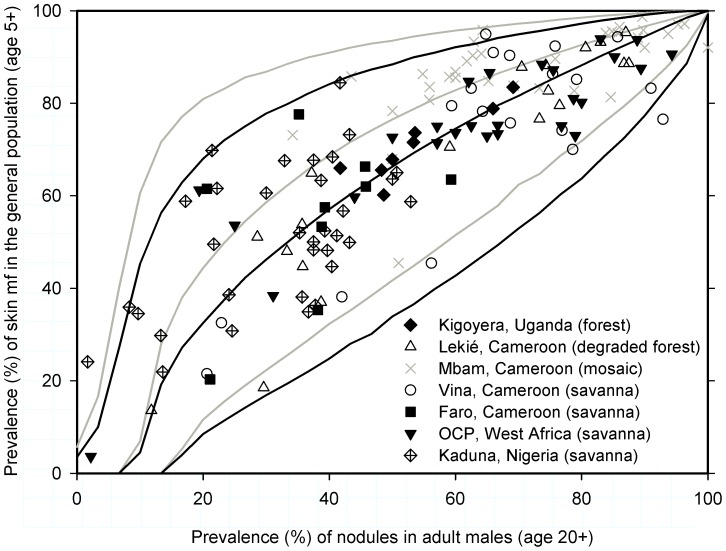
Predicted skin mf prevalence in the general population, given observed nodule prevalence in adult males. Symbols represent observed data by geographical area. Within each set of regression lines, the middle and outer lines relate to the median and 95% Bayesian credible intervals of the posterior predictive distribution, respectively (black set for areas all areas but Mbam; grey set for Mbam, the only forest-savanna mosaic area). Predictions were made assuming that nodule prevalence was based on a sample of 30 adult males.

Nodule prevalence in adult males was strongly positively correlated with mf prevalence in the general population (Table S2). There was significant geographical variation in patterns of nodule and mf prevalence; in a model without any coefficients for bioclime, the DIC increased from 1918 to 1920 when error term 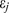 was omitted. Point estimates of 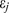 were very similar for savanna and forest areas, with the exception of Mbam, Cameroon (forest-savanna mosaic), for which mf prevalence was relatively high compared to other areas. In line with this, the model fit did not improve when a fixed effect parameter for bioclime was added to the model. However, the model fit improved significantly when modeling the difference between Mbam and all other areas as a fixed effect (DIC 1913 vs. DIC 1918), indicating that mf prevalences in Mbam were significantly higher than those in other areas (Table S2, Figure 2). After this adaptation of the model, there was still significant variation in patterns of nodule and mf prevalence between geographical areas due to other, unmeasured variables (the DIC increased to 1921 when error term 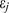 was omitted). Further, there was considerable uncertainty in the predictions for mf prevalence, based on nodule prevalence in a sample of 30 males from a hypothetical village outside the dataset (Figure 3).

**Figure 4:**
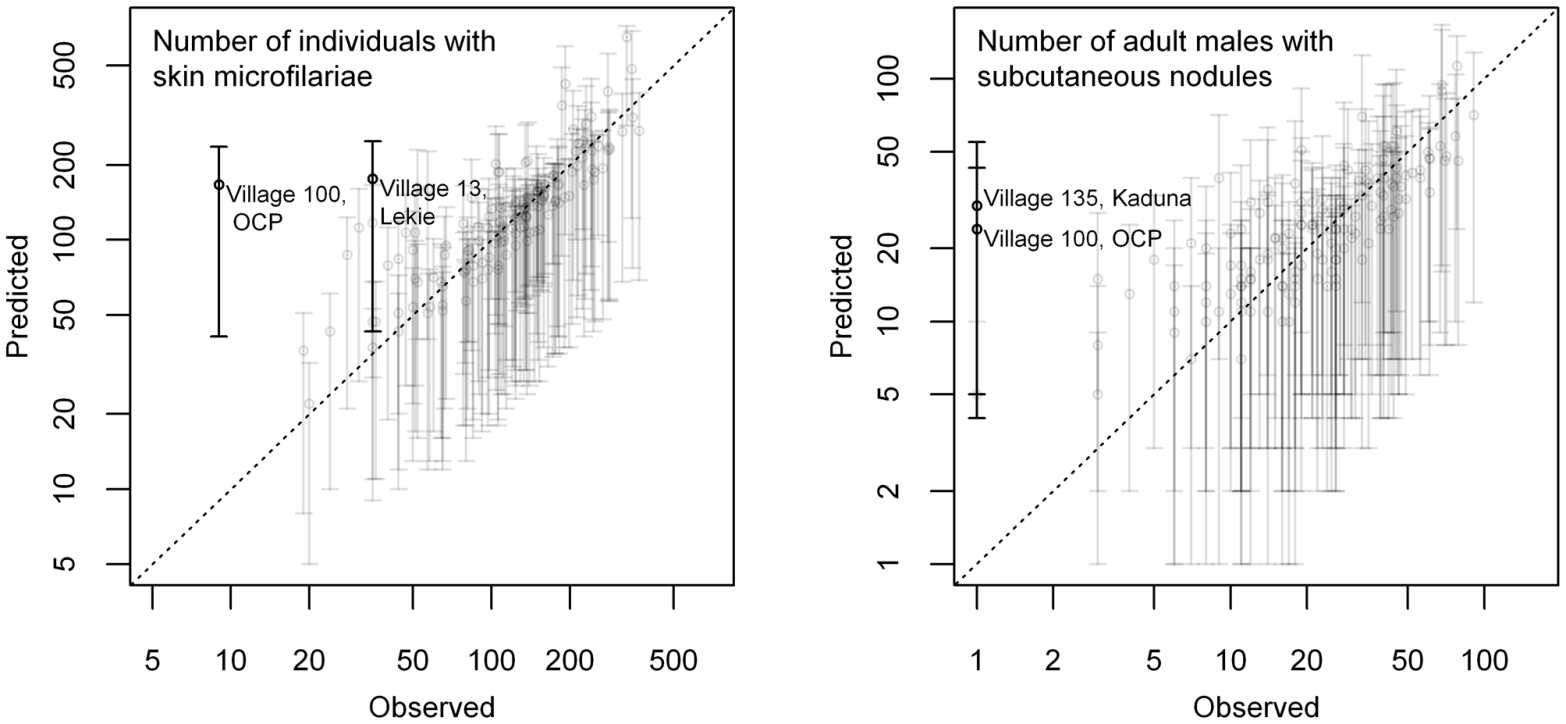
Comparison of observations (x-axis) versus model predictions (y-axis). The comparison was made by means of mixed posterior predictive checks of the numbers of individuals with detectable microfilariae in the skin and adult males with nodules. The dotted diagonal line represents the hypothetical perfect model fit. Error bars represent the 95% Bayesian prediction interval for the numbers of adult males with nodules and individuals with detectable microfilariae in the skin each village, and should intersect with the diagonal line if the model fit is good.

Mixed posterior predictive checks showed that the model fitted well to the data (Figure 4). Only three villages – all from different regions, and all with relatively low infection levels compared to other villages from the same region – deviated significantly from the model predictions.

## Discussion

We investigated the association between pre-control nodule prevalence in adult males (aged ≥20 years) and pre-control mf prevalence in the general population (aged ≥5 years). Our model is the first to examine geographical variation due to bioclime and other unmeasured variables, and to take account of measurement error in nodule prevalence. Our results show that there is a strong positive correlation between nodule and mf prevalence, but also significant variation between geographical regions, which should be taken into consideration when evaluating the prospects of elimination and the burden of disease.

Our analysis showed significant geographical variation in patterns of nodule and mf prevalence, though not related to bioclimatic zones according to the classic forest vs. savanna classification of onchocerciasis. In ‘forest’ areas – Lekié, Cameroon (degraded forest) and Kigoyera parish, Uganda (forest) – the patterns in nodule and mf prevalences did not differ much from the pattern in savanna areas. Yet, we found that mf prevalence levels in the general population were relatively higher in the only forest-savanna mosaic area (Mbam, Cameroon), while nodule prevalence in adult males levels were not significantly different. There are several possible explanations for this pattern. Most likely, the pattern in Mbam is explained by a different pattern in age-dependent exposure to black flies' bites. Both mf and nodule prevalences in individuals under the age of twenty years were relatively high in Mbam compared to the other areas in Cameroon, especially in villages with relatively low nodule prevalence in adult males (data not shown). This indicates that individuals in Mbam experience relatively high exposure levels at a young age. This might be explained by the presence of dense forest in this region with relatively few narrow open spaces, which is associated with higher dispersal of flies around the breeding sites [32]. Therefore, exposure may not be concentrated near the breeding sites, but may extend over the whole village. Related to this, exposure may be less concentrated in adults (who frequently spent time near the breeding sites, forest galleries for fishing, etc.), but may be more equally distributed over all age groups. However, dense forest may not be unique for Mbam, and may also be present in other forest areas in our data set. Therefore, we can only say that it may be important to consider age-dependent patterns in exposure to black flies' bites and their effect on transmission when translating nodule prevalence data to mf prevalence. We rule out demography and survey methods, as all mf prevalences were standardized, the mean age of the sampled men from Mbam was similar to that of men from the other Cameroonian areas, methods for skin snipping and mf enumeration were the same as in other Cameroonian areas and, in addition, even conducted by the same person (MB performed all skin snipping in Faro, Lekié, and Mbam, and 50% of skin snipping in Vina valley). Furthermore, it is also unlikely that the forest sites other than Mbam – Lekié and Kigoyera parish – harbor a savanna parasite strain (instead of the assumed forest parasite strain) as this is inconsistent with observed patterns of blindness in these areas (forest pattern) [33], [34]. Lastly, variation might have been caused by parasite characteristics not related to the classic subdivision into forest and savanna strains. Herder [35] concluded that the parasite strains circulating in the Faro and Mbam areas were related but distinct from the strains from Vina and Lekié, based on phylogenetic linkage patterns. However, this pattern was not confirmed by our analysis as the association between nodule and mf prevalence in Faro was very similar to the other areas but Mbam.

Our model could be used as a tool for assessing the prospects of elimination of onchocerciasis or the burden of onchocercal disease when pre-control nodule prevalence in adult males is the only measure of infection available (as is the case for most of Africa). With our model, an estimate of pre-control mf prevalence may be derived from pre-control nodule prevalence data. Such an estimate may be helpful for program planning, providing an indication of minimum program duration (with regard to prospects of elimination), and could be helpful in the interpretation of ongoing epidemiological parasitological surveys that rely on the skin snipping method (in terms of progress towards elimination). Prospects of elimination may be evaluated by comparing the model-derived estimate of mf prevalence to known trends of infection levels in other foci with a similar history of mass treatment, or by means of dynamic modeling of the effect of mass treatments with ivermectin using onchocerciasis transmission models such as ONCHOSIM [10], [11], [36] and others [37], [38], [39]. Progress towards elimination could be evaluated by comparing current mf prevalences with model-derived estimates of pre-control mf prevalence and predicted trends in infection levels based on dynamical modeling. Likewise, the pre-control burden of ocular and dermal morbidity in endemic areas may be estimated based on literature data on the association between mf and disease prevalence [7], [8], [9]. This would further allow assessment of the impact of control activities on population health, especially when combined with aforementioned dynamic models. If pre-control mf prevalence were to be severely underestimated or overestimated when derived from nodule prevalence data (due to measurement error and geographical variation), this may have important repercussions for the number of treatment rounds that is thought to be required to reach elimination, or the estimated burden of disease. Therefore, it is crucial to consider variation due to sample size and geographical variation in patterns of nodule and mf prevalence when doing this kind of assessment. Given the high level of variation and consequent uncertainty in the association between nodule and mf prevalence, translations should be made carefully and critically evaluated. We recommend that translations of village-level REMO data (based on samples of about 30 adult males) to mf prevalence are made based on the black lines in Figure 3 (which include uncertainty due to measurement error and geographical variation). In case of suspected high exposure of children to flies' bites, it may be more appropriate to apply the part of the model that mimics the observations in Mbam, Cameroon (grey lines in Figure 3). For areas where infection prevalence is known to be homogeneously distributed, REMO samples from multiple villages could be pooled into a more precise estimate of pre-control nodule prevalence in the area, allowing more precise prediction of the pre-control mf prevalence. In Text S1, section “Model application”, we explain in more detail how our model should be applied to convert nodule prevalence to mf prevalence (e.g. how to make predictions for a group of villages).

In conclusion, we provide a tool to convert nodule prevalence in adult males to mf prevalence in the general population, which accounts for uncertainty due to measurement error and geographical variation. This tool allows interpretation of a large amount of pre-control data on levels of infection in Africa which may a) be combined with information on coverage of mass treatment to assess the feasibility of elimination of onchocerciasis and b) enable estimation of disease burden. Furthermore, we identified significant geographical variation in mf prevalence and nodule prevalence patterns that warrants further investigation of age-dependent transmission patterns of onchocerciasis.

## Supporting Information

Table S1 Weights used to standardize mf prevalences.
(DOC)

Table S2 Parameter estimates of the model, based on Bayesian hierarchical multivariate logistic regression.
(DOC)

Text S1 Detailed description of the statistical model, and the methods used to estimate the model parameters with an explanation of how the model should be applied to data outside the current study, and providing the code that was used to specify the model in JAGS.
(PDF)
