# Supplementary material for: Onchocerciasis: The Pre-control Association between Prevalence of Palpable Nodules and Skin Microfilariae
Source: PLoS Negl Trop Dis. 2013 Apr 11;7(4):e2168. doi: 10.1371/journal.pntd.0002168 (PMC3623701; doi:10.1371/journal.pntd.0002168)
Supplement: Table S1 — Weights used to standardize mf prevalences. (DOC) [file pntd.0002168.s001.doc]

# Onchocerciasis: the pre-control association between prevalence of palpable nodules and skin microfilariae

Luc E. Coffeng,^1,a,*^ Sébastien D.S. Pion,^2,a^ Simon O’Hanlon,^3^ Simon Cousens,^4^ Adenike O. Abiose,^5^ Peter U. Fischer,^6^ Jan H.F. Remme,^7^ K.Yankum Dadzie,^8^ Michele E. Murdoch,^9^ Sake J. de Vlas,^1^ María-Gloria Basáñez,^3^ Wilma A. Stolk,^1,b^ Michel Boussinesq^2,b^

^1^ Department of Public Health, Erasmu­s MC, University Medical Center Rotterdam, P.O. Box 2040, 3000 CA Rotterdam, The Netherlands; ^2^ UMI 233, Institut de Recherche pour le Développement (IRD) and University of Montpellier 1, 911 Avenue Agropolis, BP 64501, F-34394 Montpellier cedex 5, France; ^3^ Department of Infectious Disease Epidemiology, School of Public Health, Faculty of Medicine (St Mary’s Campus), Imperial College London, Norfolk Place, London W2 1PG, UK; ^4^ Department of Epidemiology and Population Health, London School of Hygiene and Tropical Medicine, Keppel St, London WC1 E 7HT, UK;

^5^ Sightcare International, P.O. Box 29771, Secretariat Main Office, Ibadan, Oyo State, Nigeria; ^6^ Washington University School of Medicine, Infectious Disease Division, Campus Mailbox 8051, 660 South Euclid Avenue, St. Louis, MO 63110, USA;

^7^ Consultant, 120 Rue des Campanules, 01210 Ornex, France; ^8^ Consultant, P.O. Box OS-1905, Accra, Ghana; ^9^ Department of Dermatology, Watford General Hospital, Watford, Hertfordshire WD18 0HB, UK

^a,b^ These authors contributed equally to this work

* Corresponding author: Department of Public Health, Erasmus MC, University Medical Center Rotterdam, P.O. box 2040, 3000 CA Rotterdam, The Netherlands; [l.coffeng@erasmusmc.nl](mailto:l.coffeng@erasmusmc.nl), [luccoffeng@gmail.com](mailto:luccoffeng@gmail.com); tel. +31 10 70 38357, fax. +31 10 70 38474

Table S1: Weights used to standardize prevalence of microfilariae in the skin. Standardization weights were based on the reference population of the Onchocerciasis Control Programme.

| Age | Male | Female |
| --- | --- | --- |
| 5–9 | 0.091 | 0.078 |
| 10–14 | 0.090 | 0.077 |
| 15–29 | 0.129 | 0.138 |
| 30–49 | 0.123 | 0.146 |
| ≥ 50 | 0.063 | 0.064 |
