# Supplementary material for: Onchocerciasis: The Pre-control Association between Prevalence of Palpable Nodules and Skin Microfilariae
Source: PLoS Negl Trop Dis. 2013 Apr 11;7(4):e2168. doi: 10.1371/journal.pntd.0002168 (PMC3623701; doi:10.1371/journal.pntd.0002168)
Supplement: Table S2 — Parameter estimates of the model, based on Bayesian hierarchical multivariate logistic regression. (DOC) [file pntd.0002168.s002.doc]

# Onchocerciasis: the pre-control association between prevalence of palpable nodules and skin microfilariae

Luc E. Coffeng,^1,a,*^ Sébastien D.S. Pion,^2,a^ Simon O’Hanlon,^3^ Simon Cousens,^4^ Adenike O. Abiose,^5^ Peter U. Fischer,^6^ Jan H.F. Remme,^7^ K.Yankum Dadzie,^8^ Michele E. Murdoch,^9^ Sake J. de Vlas,^1^ María-Gloria Basáñez,^3^ Wilma A. Stolk,^1,b^ Michel Boussinesq^2,b^

^1^ Department of Public Health, Erasmu­s MC, University Medical Center Rotterdam, P.O. Box 2040, 3000 CA Rotterdam, The Netherlands; ^2^ UMI 233, Institut de Recherche pour le Développement (IRD) and University of Montpellier 1, 911 Avenue Agropolis, BP 64501, F-34394 Montpellier cedex 5, France; ^3^ Department of Infectious Disease Epidemiology, School of Public Health, Faculty of Medicine (St Mary’s Campus), Imperial College London, Norfolk Place, London W2 1PG, UK; ^4^ Department of Epidemiology and Population Health, London School of Hygiene and Tropical Medicine, Keppel St, London WC1 E 7HT, UK;

^5^ Sightcare International, P.O. Box 29771, Secretariat Main Office, Ibadan, Oyo State, Nigeria; ^6^ Washington University School of Medicine, Infectious Disease Division, Campus Mailbox 8051, 660 South Euclid Avenue, St. Louis, MO 63110, USA;

^7^ Consultant, 120 Rue des Campanules, 01210 Ornex, France; ^8^ Consultant, P.O. Box OS-1905, Accra, Ghana; ^9^ Department of Dermatology, Watford General Hospital, Watford, Hertfordshire WD18 0HB, UK

^a,b^ These authors contributed equally to this work

* Corresponding author: Department of Public Health, Erasmus MC, University Medical Center Rotterdam, P.O. box 2040, 3000 CA Rotterdam, The Netherlands; [l.coffeng@erasmusmc.nl](mailto:l.coffeng@erasmusmc.nl), [luccoffeng@gmail.com](mailto:luccoffeng@gmail.com); tel. +31 10 70 38357, fax. +31 10 70 38474

Table S2. Model parameter estimates from Bayesian hierarchical multivariate logistic regression of infection prevalence data. The model predicts the joint distribution of prevalence of nodules in adult males (age ≥20) and presence of microfilariae (mf) in the skin of the general population (age ≥5).

| **Parameter^a^** | **Interpretation** | **Median^b^** | **Lower bound^c^** | **Upper bound^d^** |
| --- | --- | --- | --- | --- |
| $\text{logit}^{\text{-1}}\left(\beta_{0,m=1}\right)$ | Average fraction of general population with mf in the skin (excluding Mbam) | 0.68 | 0.55 | 0.78 |
| $\text{logit}^{\text{-1}}\left(\beta_{0,m=2}\right)$ | Average fraction of adult males with onchocercal nodules (excluding Mbam) | 0.51 | 0.36 | 0.67 |
| $\exp\left(\beta_{1,m=1}\right)$ | Odds ratio of presence of mf in the skin in Mbam compared to other areas | 4.17 | 1.04 | 16.69 |
| $\exp\left(\beta_{1,m=2}\right)$ | Odds ratio of presence of nodules in Mbam compared to other areas | 2.69 | 0.45 | 14.57 |
| $\sigma_{ij,m=1}$ | Standard deviation of log odds of presence of mf within geographical areas | 0.98 | 0.87 | 1.11 |
| $\sigma_{ij,m=2}$ | Standard deviation of log odds of presence of nodules within geographical areas | 0.89 | 0.77 | 1.03 |
| $\rho_{ij}$ | Correlation of log odds of presence of nodules and mf within geographical areas | 0.84 | 0.77 | 0.90 |
| $\sigma_{j,m=1}$ | Standard deviation of average log odds of presence of mf between geographical areas | 0.55 | 0.22 | 1.24 |
| $\sigma_{j,m=2}$ | Standard deviation of average log odds of presence of nodules between geographical areas | 0.69 | 0.31 | 1.50 |
| $\rho_{j}$ | Correlation of average log odds of presence of nodules and mf between geographical areas | 0.88 | 0.28 | 1.00 |
| specificity | One minus the probability of misclassifying a subcutaneous nodule as onchocercal | 0.99 | 0.98 | 1.00 |
| ^a^ For ease of interpretation, parameter estimates have been transformed to an intuitive scale, where possible (inverse logit transformation for intercepts and exponents for other fixed effects parameters). See Appendix A for a detailed description of the model and its parameters.  ^b^ Median of posterior distribution.  ^c^ Defined as the 2.5^th^ percentile of the posterior distribution.  ^d^ Defined as the 97.5^th^ percentile of the posterior distribution. | | | | |
