# Supplementary material for: Onchocerciasis: The Pre-control Association between Prevalence of Palpable Nodules and Skin Microfilariae
Source: PLoS Negl Trop Dis. 2013 Apr 11;7(4):e2168. doi: 10.1371/journal.pntd.0002168 (PMC3623701; doi:10.1371/journal.pntd.0002168)
Supplement: Text S1 — Detailed description of the statistical model, and the methods used to estimate the model parameters with an explanation of how the model should be applied to data outside the current study, and providing the code that was used to specify the model in JAGS. (PDF) [file pntd.0002168.s003.pdf]

# Onchocerciasis: the pre-control association between prevalence of palpable nodules and skin microfilariae

Luc E. Coffeng,[1,a,*] Sébastien D.S. Pion,[2,a] Simon O'Hanlon,[3] Simon Cousens,[4] Adenike O. Abiose,[5] Peter U. Fischer,[6,7] Jan H.F. Remme,[8] K.Yankum Dadzie,[9] Michele E. Murdoch,[10] Sake J. de Vlas,[1] María-Gloria Basáñez,[3] Wilma A. Stolk,[1,b] Michel Boussinesq[2,b]

[1] Department of Public Health, Erasmus MC, University Medical Center Rotterdam, P.O. Box 2040, 3000 CA Rotterdam, The Netherlands; [2] UMI 233, Institut de Recherche pour le Développement (IRD) and University of Montpellier 1, 911 Avenue Agropolis, BP 64501, F-34394 Montpellier cedex 5, France; [3] Department of Infectious Disease Epidemiology, School of Public Health, Faculty of Medicine (St Mary's Campus), Imperial College London, Norfolk Place, London W2 1PG, UK; [4] Department of Epidemiology and Population Health, London School of Hygiene and Tropical Medicine, Keppel St, London WC1 E 7HT, UK; [5] Sightcare International, P.O. Box 29771, Secretariat Main Office, Ibadan, Oyo State, Nigeria; [6] Washington University School of Medicine, Infectious Disease Division, Campus Mailbox 8051, 660 South Euclid Avenue, St. Louis, MO 63110, USA; [7] Bernhard Nocht Institute for Tropical Medicine, Hamburg, Germany; [8] Consultant, 120 Rue des Campanules, 01210 Ornex, France; [9] Consultant, P.O. Box OS-1905, Accra, Ghana; [10] Department of Dermatology, Watford General Hospital, Watford, Hertfordshire WD18 0HB, UK

[a,b] These authors contributed equally to this work
* Corresponding author: Department of Public Health, Erasmus MC, University Medical Center Rotterdam, P.O. box 2040, 3000 CA Rotterdam, The Netherlands; l.coffeng@erasmusmc.nl, luccoffeng@gmail.com; tel. +31 10 70 38357, fax. +31 10 70 38474

# Text S1

## Contents

Model description ... 1
Parameter estimation ... 3
Model application ... 5
Model specification in JAGS ... 7
References ... 8

## Model description

The current study considers spatially clustered, bivariate binomially distributed data. For analysis of spatial data, there are geostatistical techniques that take account of the spatial correlation. These techniques require knowledge of the geographical coordinates of the data points, which were not fully available in our case. Instead, we used an ordinary hierarchical approach to model the data to account for spatial correlation.

There are several popular alternatives for modeling bivariate binomially distributed data. Multivariate probit regression models have been proposed. These models are convenient in terms of computational requirements because they only quantify the correlation between observations, leaving variance at the lowest level of the data unidentified (but accounted for) [1]. Depending on the researcher's objective, this can be considered an advantage (computationally less demanding) or a drawback (part of the model remains unidentified). Another drawback is that the interpretation of probit models may be less intuitive to some, if not many researchers. As a concession to these arguments, a

reparameterization of the multivariate logistic regression model has been proposed, which like the probit model leaves the variance at the lowest level unidentified [1,2]. However, this reparameterized logistic model requires a customized sampling algorithm for efficient Markov chain Monte Carlo sampling. In our case, we chose for intuitive interpretation, quantification of all variances, and the use of the freely available and widely implemented Gibbs sampling algorithm, leading to the choice of generalizing the familiar logistic model as described below.

The multivariate model used in this study is an extension of the hierarchical logistic regression model $\text{logit}(\pi_{ij}(y_{ij} = k_{ij} \mid \mathrm{X}_{ij}, \beta, n_{ij})) = \mathrm{X}_{ij}^T \beta + \varepsilon_{ij} + \varepsilon_j$, where $\pi_{ij}$ is the probability of finding $k$ cases of the binomially distributed outcome $y$ in $n$ individuals from the *i*-th unit in the *j*-th cluster, conditional on a set of observed covariates $\mathrm{X}_{ij}$ and a set of model parameters $\beta$. The error terms $\varepsilon_{ij}$ and $\varepsilon_j$ represent the variation within and between the *j* clusters of observation, respectively. We extended this model to simultaneously predict *m* binary outcomes, leading to $\text{logit}(\pi_{ij,m}(y_{ij,m} = k_{ij,m} \mid \mathrm{X}_{ij}, \beta_m, n_{ij,m})) = \mathrm{X}_{ij}^T \beta_m + \varepsilon_{ij} + \varepsilon_j$, where $\pi_{ij,m}$ is the probability of observing $k$ cases of the *m*-th outcome (*m* = 1: presence of microfilariae in the skin; *m* = 2: presence of nodules in adult males) among $n_m$ observed individuals from the *i*-th unit (village) in the *j*-th cluster (geographical area). Here, the error terms $\varepsilon_{ij}$ and $\varepsilon_j$ each consist of *m* components representing the variation in log odds of each of the *m* outcomes within and between the *j* clusters of observations. For each observation, there is a set of observed covariates $\mathrm{X}_{ij}$ (bioclime), and for each of the *m* predicted binary outcomes we have a set model parameters $\beta_m$. In our case, the intercepts $\beta_{0,m=1}$ and $\beta_{0,m=2}$ represent the mean log odds of presence of mf and nodule in the data, respectively. The parameters $\beta_{1,m=1}$ and $\beta_{1,m=2}$ represent the log odds ratio of observing presence of microfilariae in the skin and subcutaneous onchocercal nodules in a certain bioclime, respectively, relative to a reference bioclime (multiple sets of such parameter can be added to stratify the analysis by multiple bioclimes and/or other characteristics). Correlation between onchocercal nodule and mf prevalence was modeled by assuming multivariate normal (MVN) distributions for the error terms: $\varepsilon_{ij} \sim MVN(\mu_{\varepsilon_{ij}}, \Sigma_{\varepsilon_{ij}})$ and $\varepsilon_j \sim MVN(\mu_{\varepsilon_j}, \Sigma_{\varepsilon_j})$, with $\mu_{\varepsilon_{ij}} = \mu_{\varepsilon_j} = (0,0)$. Here, $\Sigma_{\varepsilon_{ij}}$ and $\Sigma_{\varepsilon_j}$ are variance-covariance matrices with size *m* x *m*, containing along the diagonal the marginal variances of the errors for the log odds of

presence of nodules and mf within ($\sigma^2_{ij,m=1}$ and $\sigma^2_{ij,m=2}$) and between ($\sigma^2_{j,m=1}$ and $\sigma^2_{j,m=2}$) the *j* clusters of observations. The off-diagonal positions of $\Sigma_{\varepsilon_{ij}}$ and $\Sigma_{\varepsilon_j}$ hold the covariances $\sigma_{ij,m=1;m=2}$ and $\sigma_{j,m=1;m=2}$ of error terms within and between the *j* clusters, respectively. The correlation between log odds of presence of nodules and mf at village-level $\rho_{ij}$ was derived by $\frac{\sigma_{ij,m=1;m=2}}{\sigma_{ij,m=1}\sigma_{ij,m=2}}$. Correlation $\rho_j$ was derived in a similar fashion, and can be interpreted in two ways; 1) together with variances $\sigma^2_{j,m=1}$ and $\sigma^2_{j,m=2}$, $\rho_j$ represents how the association between onchocercal nodule and mf prevalences varies between geographical regions due to e.g. environmental factors and surveys methods (analogous to linear regression models with a random intercept); 2) $\rho_j$ is the correlation between the mean log odds of presence of nodules and mf in a geographical area (as defined for the data in this study).

## Parameter estimation

Model parameters were estimated assuming non-informative prior distributions. For fixed effects parameters $\beta_m$, we assumed independent normal prior distributions $N_{\beta_m}(0,1000)$. The village-level variance-covariance matrix $\Sigma_{\varepsilon_{ij}}$ was estimated assuming a scaled Wishart prior distribution $W_m(R,k)$ for its inverse $\Sigma^{-1}_{\varepsilon_{ij}}$, where $R$ is the *m* x *m* identity matrix $I_m$, and $k$ is the number of degrees of freedom (set to 3, effectively assuming uniform prior information on $\rho_{ij}$). To maximize the speed of model convergence, the variance-covariance matrix $\Sigma_{\varepsilon_j}$ for differences between geographical areas was hierarchically centered around fixed effects $\beta_m$, and was estimated assuming independent uniform prior distributions for the correlation ($\rho_j \sim U(-1,1)$), and standard deviations ($\sigma_{j,m} \sim U(0,10)$ or $\sigma_{j,m} \sim U(0,100)$), in line with previous recommendations for estimating hyperparameters [3]. The prior distribution for minimum sensitivity of nodule palpation (at low endemicity levels) was defined as being uniform between 60% and 100%. The prior distribution for specificity of nodule palpation was defined as being uniform between 98% and 100%.

**Figure A1. Autocorrelation plots of Monte Carlo samples for nine parameters.** In this study, autocorrelation was initially high for some parameters, indicating that the Gibbs sampling algorithm was slow in exploring the posterior distribution of these parameters. Autocorrelation was reduced by storing only every 20th Monte Carlo sample and running 200,000 iterations (after discarding an initial 200,000 iterations for burn-in), for which the results are shown here. After this, autocorrelation of Monte Carlo samples was similarly low for all parameters and Markov chains.

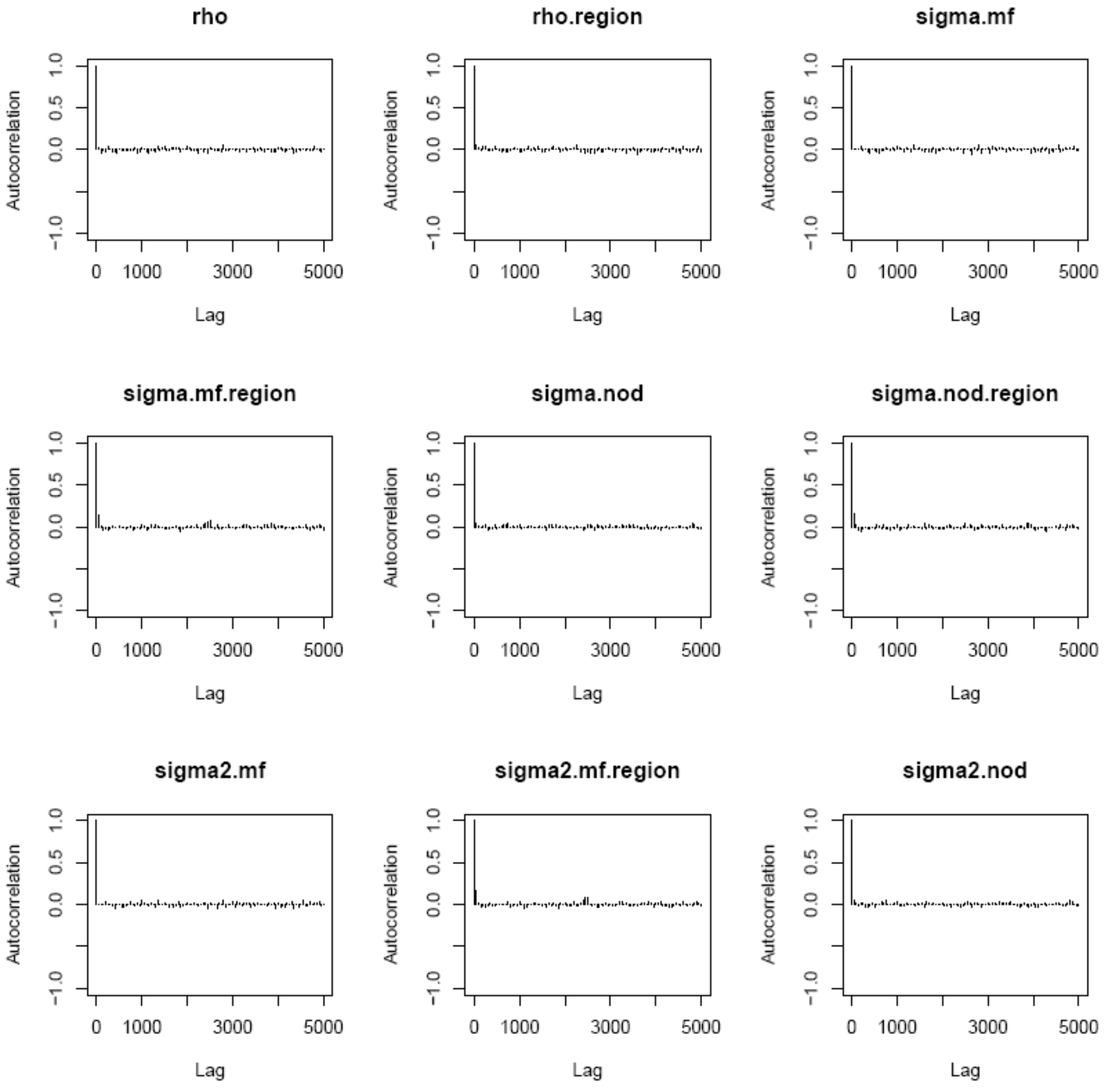


Model parameters were estimated using four Markov chains with each 400,000 Monte Carlo simulations. For each chain, the first 200,000 of the saved simulations were considered as burn-in simulations and discarded. Such a number of simulations was necessary as the Gibbs sampler explored the joint posterior distribution of parameters slowly, indicated by high autocorrelation of Monte Carlo samples. To save storage space, only every 20th Monte Carlo sample was stored,

effectively reducing autocorrelation (Figure A1). The effective amount of simulations per parameter was 40,000 (sum of four Markov chains). The point estimate for each parameter and prediction were taken to be the median of the 40,000 simulations. Ninety five percent Bayesian credible intervals were calculated as the 2.5$^{th}$ and 97.5$^{th}$ percentiles of the simulations.

Model convergence was assessed by checking whether the four Markov chains converged to the same posterior distribution for each parameter, based on Gelman and Rubin's convergence diagnostic, the potential scale reduction factor (which should be below 1.1; i.e. a posterior credible interval of a parameter estimate should not become more than 10% narrower if more Monte Carlo samples were drawn) [4]. Because this diagnostic test requires that starting values for each parameter in each Markov Chain are over-dispersed with respect to the true posterior distribution of a parameter, we assigned heavily over-dispersed initial values to each of the model parameters in each chain (e.g. the initial values 1, 10, 50, and 100 for a parameter with an uninformative normal distribution as prior, one value for each Markov chain). In our simulations, the potential scale reduction factor was at or below 1.001 for all parameters. Furthermore, we checked that all Markov chains arrived in the joint posterior distribution of parameter values, as determined by means of Geweke's test, which compares the distribution of the first 10% and last 50% of the Monte Carlo samples within a chain [5]. We also checked that Monte Carlo errors were small, relative to the point estimate of each parameter (difference of at least factor 100 – 1000).

## Model application

Given estimates of $\beta_m$, $\Sigma_{\varepsilon_{ij}}$, and $\Sigma_{\varepsilon_j}$, we estimated the conditional distribution of mf prevalence $\pi^*_{ij,m=1} \mid \pi^*_{ij,m=2}$ in a hypothetical village *i* from an unspecified region *j* outside the dataset, given an estimate of the 'true' onchocercal nodule prevalence in adult males, corrected for misclassification of nodules, in the same hypothetical village (assuming this is exactly known). Given that we are working with multivariate normal distributions, the conditional distribution for $\pi^*_{ij,m=1} \mid \pi^*_{ij,m=2}$ can be described as

$$\text{logit}\left(\pi^*_{ij,m=1} \mid \pi^*_{ij,m=2}\right) \sim N\left( X^T_{ij}\beta^*_{m=1} + \frac{\sigma_{ij,m=1}}{\sigma_{ij,m=2}}\rho_{ij}\left(\text{logit}\left(\pi^*_{ij,m=2}\right) - X^T_{ij}\beta_{m=2}\right), \left(1-\rho^2_{ij}\right)\sigma^2_{ij,m=1}\right),$$ where

$\beta^*_{m=1} \sim N\left(\beta_{m=1}, \left(1-\rho^2_j\right)\sigma^2_{j,m=1}\right)$. To include uncertainty about nodule prevalence $\pi^*_{ij,m=2}$ in the

prediction of mf prevalence $\pi^{*}_{ij,m=1}$, we simulated values from the estimated distribution of $\text{logit}\left(\pi^{*}_{ij,m=2}\right)$, and fed these into the distribution for $\text{logit}\left(\pi^{*}_{ij,m=1} \mid \pi^{*}_{ij,m=2}\right)$, from which values for $\pi^{*}_{ij,m=1}$ were then simulated. As this was done while simultaneously estimating the values of model parameters by means of Markov Chain Monte Carlo sampling, all uncertainty in the model parameter estimates was carried through to the final predictions for mf prevalence in hypothetical villages.

It should be noted that the procedure described above produces predictions pertaining to individual villages only, and therefore will produce predictions containing a great deal of uncertainty. The amount of uncertainty would be substantially lower if predictions were made based on larger samples of adult males, or when made for the mean prevalence of infection in a group of villages. However, a prediction for a mean prevalence would ignore possible heterogeneity in infection prevalences between villages, which may lead to overly optimistic estimates when e.g. when assessing prospects of elimination (the most highly endemic village will determine the required duration of an intervention, not the mean prevalence in a region). Nevertheless, if such predictions are made (e.g. for a group of villages with known similar levels of infection), or concerning many villages (theoretically an infinite number of villages), the mean mf prevalence is described by $\mathrm{X}_{ij}^{T}\beta^{*}_{m=1} + \frac{\sigma_{ij,m=1}}{\sigma_{ij,m=2}}\rho_{ij}\left(\text{logit}\left(\pi^{*}_{ij,m=2}\right) - \mathrm{X}_{ij}^{T}\beta_{m=2}\right)$, where $\pi^{*}_{ij,m=2}$ is the mean nodule prevalence in the group of villages (including uncertainty related to overall sample size). However, usually the number of sampled villages is not very high (<1,000, meaning that the denominator of the standard error of the mean is <30, approx. the square root of 1,000), and one should therefore simulate the mf prevalence separately for every village, sampling village-level error independently for every village, and sampling the region-level error simultaneously for all villages. Then, for every set of many repeated simulations (i.e. a set consisting of one simulation for each village), the investigator can calculate the mean or any other summary statistic of the level of infection in the group of villages (e.g. range or variance), arriving at a distribution for the estimated mean or another summary statistic for mf prevalence in a group of villages.

## Model specification in JAGS

```
for (i in 1:N) {
	# Likelihood of nodule data
	k.nod[i] ~ dbin(sens.nod.p[i] * p.nod[i] + (1-spec.nod)*(1-p.nod[i]), n.nod[i])
	sens.nod.p[i] <- sens.nod + (1-sens.nod)*p.nod[i]
	logit(p.nod[i]) <- B0[region[i],1] + e.vill[i,1]

	# Likelihood of mf data
	k.mf[i] ~ dbin(p.mf[i],n.mf[i])
	logit(p.mf[i]) <- B0[region[i],2] + e.vill[i,2]

	# Correlation of nodule and mf data with regions
	e.vill[i,1] <- e.vill.raw[i,1] * xi.nod
	e.vill[i,2] <- e.vill.raw[i,2] * xi.mf
	e.vill.raw[i,1:2] ~ dmnorm(Mu,Sigma2.inv.raw)
}

# Priors for fixed effects
sens.nod ~ dunif( [some value] ,1.0) # [some value] = 0.6, 0.8, or 1.0 (final model)
spec.nod ~ dunif(0.98 ,1)
b0.nod ~ dnorm(0,0.001)
b0.mf ~ dnorm(0,0.001)
mbam.nod ~ dnorm(0,0.001)
mbam.mf ~ dnorm(0,0.001)

# Uniform prior for correlation and marginal standard deviations
# of hierarchically centered random region effects
for (j in 1:7) {
	B0[j,1:2] ~ dmnorm(Mu.region[j,1:2],Sigma2.region.inv)
	Mu.region[j,1] <- b0.nod + mbam.nod*equals(j,2)
	Mu.region[j,2] <- b0.mf + mbam.mf*equals(j,2)
}
Sigma2.region.inv <- inverse(Sigma2.region)
Sigma2.region[1,1] <- sigma2.nod.region
Sigma2.region[2,2] <- sigma2.mf.region
Sigma2.region[1,2] <- covar.nod.mf.region
Sigma2.region[2,1] <- covar.nod.mf.region
covar.nod.mf.region <- rho.region * sigma.nod.region * sigma.mf.region
sigma2.nod.region <- pow(sigma.nod.region,2)
sigma2.mf.region <- pow(sigma.mf.region,2)
sigma.nod.region ~ dunif(0,10)
sigma.mf.region ~ dunif(0,10)
rho.region ~ dunif(-1,1)

# Scaled inverse Wishart prior for random village effects
Sigma2.inv.raw ~ dwish(R,scale)
xi.nod ~ dunif(0,100)
xi.mf ~ dunif(0,100)
Sigma2.raw <- inverse(Sigma2.inv.raw)
sigma.nod <- pow(Sigma2.raw[1,1],0.5) * xi.nod
sigma.mf <- pow(Sigma2.raw[2,2],0.5) * xi.mf
rho <- Sigma2.raw[1,2]/sqrt(Sigma2.raw[1,1]*Sigma2.raw[2,2])

Sigma2[1,1] <- pow(sigma.nod,2)
Sigma2[1,2] <- rho * sigma.nod * sigma.mf
Sigma2[2,1] <- Sigma2[1,2]
Sigma2[2,2] <- pow(sigma.mf,2)
```

```
# Predictions for REMO samples
sigma2.mf.REMO <- (1 - rho^2) * pow(sigma.mf,2)
sigma2.mf.REMO.region <- (1 - rho.region^2) * sigma2.mf.region
tau.mf.REMO <- pow(sigma2.mf.REMO,-1)
tau.mf.REMO.region <- pow(sigma2.mf.REMO.region,-1)

for (k in 1:N.REMO) {
	# Hypothetical REMO village: nodule prevalence
	k.nod.REMO[k] ~ dbin(sens.nod.p.REMO[k] * p.nod.REMO[k] +
					(1-spec.nod)*(1-p.nod.REMO[k]),n.nod.REMO[k])
	sens.nod.p.REMO[k] <- sens.nod + (1-sens.nod)*p.nod.REMO[k]
	logit(p.nod.REMO[k]) <- b.nod.REMO[k]
	b.nod.REMO[k] ~ dnorm(0,0.001)

	# Hypothetical REMO village: mf prevalence (non-mosaic)
	logit(p.mf.REMO.vill[k]) <- b.mf.REMO.vill[k]
	b.mf.REMO.vill[k] ~ dnorm(b0.mf.REMO.region[k],tau.mf.REMO)
	b0.mf.REMO.region[k] <- b0.mf.REMO.intercept[k] +
				(sigma.mf/sigma.nod) * rho * (b.nod.REMO[k] - b0.nod)
	b0.mf.REMO.intercept[k] ~ dnorm(b0.mf,tau.mf.REMO.region)

	# Hypothetical REMO village: mf prevalence (mosaic)
	logit(p.mf.REMO.vill.mosaic[k]) <- b.mf.REMO.mosaic.vill[k]
	b.mf.REMO.mosaic.vill[k] ~ dnorm(b0.mf.REMO.mosaic.region[k],tau.mf.REMO)
	b0.mf.REMO.mosaic.region[k] <- b0.mf.REMO.mosaic.intercept[k] +
			(sigma.mf/sigma.nod) * rho * (b.nod.REMO[k] - (b0.nod + mbam.nod))
	b0.mf.REMO.mosaic.intercept[k] ~ dnorm((b0.mf + mbam.mf),tau.mf.REMO.region)
}
```

## References


1. O'Brien SM, Dunson DB (2004) Bayesian multivariate logistic regression. Biometrics 60: 739-746.

2. Ovaskainen O, Hottola J, Siitonen J (2010) Modeling species co-occurrence by multivariate logistic regression generates new hypotheses on fungal interactions. Ecology 91: 2514-2521.

3. Gelman A (2006) Prior distributions for variance parameters in hierarchical models. Bayesian Analysis 1: 515-533.

4. Gelman A, Rubin DB (1992) Inference from iterative simulation using multiple sequences. Statistical Science 7: 457-511.

5. Geweke J (1991) Evaluating the accuracy of sampling-based approaches to the calculation of posterior moments. In: Bernado J, Berger J, Dawid A, Smith A, editors. Bayesian statistics 4. Oxford, UK: Clarendon Press.
